# TdIF1 Recognizes a Specific DNA Sequence through Its Helix-Turn-Helix and AT-Hook Motifs to Regulate Gene Transcription

**DOI:** 10.1371/journal.pone.0066710

**Published:** 2013-07-10

**Authors:** Takashi Kubota, Osamu Koiwai, Katsutoshi Hori, Nobuhisa Watanabe, Kotaro Koiwai

**Affiliations:** 1 Department of Applied Biological Science, Faculty of Science and Technology, Tokyo University of Science, Noda, Chiba, Japan; 2 Department of Biotechnology, Graduate School of Engineering, Nagoya University, Nagoya, Aichi, Japan; 3 NUSR, Nagoya University, Nagoya, Aichi, Japan; Université Paris-Diderot, France

## Abstract

TdIF1 was originally identified as a protein that directly binds to DNA polymerase TdT. TdIF1 is also thought to function in transcription regulation, because it binds directly to the transcriptional factor TReP-132, and to histone deacetylases HDAC1 and HDAC2. Here we show that TdIF1 recognizes a specific DNA sequence and regulates gene transcription. By constructing TdIF1 mutants, we identify amino acid residues essential for its interaction with DNA. An *in vitro* DNA selection assay, SELEX, reveals that TdIF1 preferentially binds to the sequence 5′-GNTGCATG-3′ following an AT-tract, through its Helix-Turn-Helix and AT-hook motifs. We show that four repeats of this recognition sequence allow TdIF1 to regulate gene transcription in a plasmid-based luciferase reporter assay. We demonstrate that TdIF1 associates with the RAB20 promoter, and RAB20 gene transcription is reduced in TdIF1-knocked-down cells, suggesting that TdIF1 stimulates RAB20 gene transcription.

## Introduction

TdT interacting factor 1 (TdIF1), encoded by *DNTTIP1*, was first identified as a protein that directly binds to terminal deoxynucleotidyltransferase (TdT) [1]. TdT contributes to the diversity of immunoglobulins and T-cell receptors in lymphocytes [2], [3]. TdIF1 negatively regulates TdT activity [1], [4], [5] and controls TdT degradation through the Bood POZ-containing gene type-2 (BPOZ-2)-mediated ubiquitin proteasome system [6], [7]. These previous studies showed that TdIF1 controls TdT in lymphocytes at the post-translational level.

TdT is expressed specifically in lymphocytes, but TdIF1 is expressed ubiquitously suggesting it has additional biological functions. TdIF1 orthologues are even found in invertebrates, such as *Caenorhabditis elegans* (*C. elegans*), which do not possess a TdT gene [5], [8], strongly suggesting that TdIF1 functions in non-lymphoid as well as lymphoid cells. Several lines of evidence implicate TdIF1 in controlling transcription. Human TdIF1 is reported to bind to TReP-132 (also known as TRERF1), a transcriptional co-activator of steroidogenic factor 1, which induces p450scc gene expression in steroid-hormone-producing cells [4], [9]. In addition, in M-phase cells human TdIF1 associates with histone deacetylases HDAC1 and HDAC2 in a multisubunit complex [10], that may act in transcriptional regulation. In *C. elegans,* the TdIF1 orthologue also associates with the TReP-132 orthologue and with histone deacetylase HDA-2, and is suggested to act downstream of cGMP-dependent protein kinase to regulate gene expression [8].

TdIF1 is a 37-kDa DNA-binding protein that contains three DNA-binding regions: within residues 1–75, an AT-hook domain between residues 159–173, and a predicted helix-turn-helix (HTH) region between residues 184–243 [5]. The AT-hook, which was first described in the high-mobility-group non-histone chromosomal protein HMGA, binds to AT-tracts in the minor groove of DNA [11], [12]. The HTH is a short structural motif consisting of a first α-helix, a connecting turn, and a second helix, which generally recognizes a specific DNA sequence [13]. While TdIF1 binds to AT-tracts through the AT-hook [5], no evidence has been reported for recognition of a specific DNA sequence by the predicted HTH of TdIF1.

Here we show that basic amino acids present in the three DNA-binding regions of TdIF1 (residues 1–75, AT-hook, and HTH) are required for its DNA binding. Using an *in vitro* binding sequence selection assay (SELEX), and competitive electrophoretic mobility shift assay (EMSA), we find that TdIF1 preferentially binds to the specific DNA sequence 5′-GNTGCATG-3′ where it follows AT-tracts, through its AT-hook and HTH domains. Furthermore, we showed that these recognition sequences allow TdIF1 to up-regulate gene transcription in a luciferase reporter system. Finally, we show that TdIF1 associates with the promoter region of the RAB20 gene to regulate its transcription.

## Results

### Basic amino acid residues in three DNA-binding regions of TdIF1 important for its DNA binding

**Figure 1:**
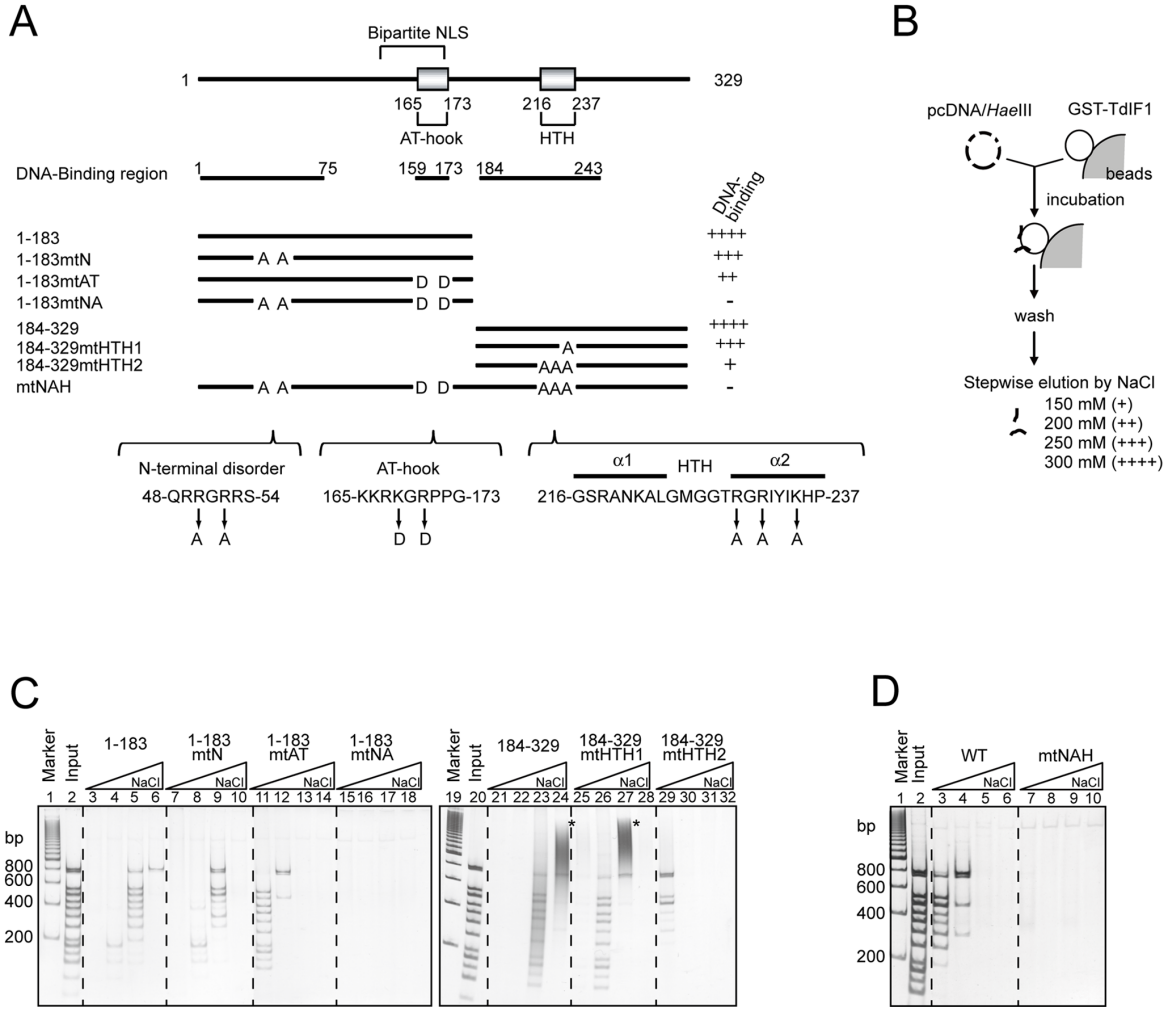
Basic amino acids in residues 1–75, an AT-hook, and an HTH of TdIF1 are required for its DNA-binding activity. (A) Schematic representation of TdIF1 mutants and summary of their DNA binding activity. DNA binding regions which are previously determined [5] are shown below the schematic representation of TdIF1. “DNA-binding” shows summary of GST pull-out assay shown in panels C and D: DNA fragments were held by TdIF1 or TdIF1 mutants until elution in buffer containing 300 mM NaCl (++++), 250 mM (+++), 200 mM (++) or 150 mM (+), or DNA fragments were not held (−). NLS, nuclear localization signal. (B) Schematic flowchart of GST pull-out assay. (C) DNA-binding activities of TdIF1 mutants. *Hae* III-digested pcDNA3.1 plasmid was incubated with TdIF1 mutants, and then DNA fragments bound to TdIF1 mutants were sequentially eluted with buffer A containing 150, 200, 250, or 300 mM NaCl, separated by PAGE, and detected by silver staining. Lanes 1 and 19 contained a 200-bp DNA ladder marker. Lanes 2 and 20 contained 1/5 of the amount of pcDNA3.1/*Hae* III used in the reaction. Asterisk indicates higher molecular weight bands, which are probably a complex of DNA and protein eluted. (D) DNA-binding activity of full-length TdIF1 containing point mutations. DNA fragments bound to TdIF1 mutants were sequentially eluted with buffer containing 200, 250, 300, or 350 mM NaCl, and analysed as in (C).

We previously showed that TdIF1 binds to dsDNA through three regions: residues 1–75, an AT-hook spanning residues 159–173, and residues 184–243 containing a predicted HTH [5]. To identify the amino acid residues that bind to DNA, we constructed a series of TdIF1 mutants (Figure 1A). Residues 48–54 are predicted by DISOPRED to produce a disordered, structurally flexible region that could potentially bind DNA or proteins [14], so in a C-terminally truncated TdIF1 protein we replaced R50 and R52 with alanines (1–183mtN). We also introduced two missense mutations in the AT hook region (1–183mtAT), similar to mutations made in AT-hook protein HMGA [15]. To determine whether the predicted HTH binds to DNA, in an N-terminally truncated TdIF1 we replaced K235 with alanine (184–329mtHTH1). K235 lies in the second helix of the HTH motif and is conserved from *C. elegans* to humans. We also replaced other two basic amino acid residues in the second helix with alanines (184–329mtHTH2), because the second helix in an HTH is generally considered to recognize a specific DNA sequence [13] and positively charged amino acids may contact DNA phosphates [16]. Finally, we constructed a mutant mtNAH, with all these point mutations in the full-length TdIF1.

To examine the DNA-binding activity of these mutants, we performed GST pull-out assays (Figure 1B) [5]. DNA fragments produced by digesting the pcDNA3.1 plasmid with *Hae* III were incubated with GST-fused TdIF1 immobilized on glutathione Sepharose beads. The DNA fragments that bound to TdIF1 were sequentially eluted with buffer containing 150–300 mM NaCl and analysed by PAGE. This assay allows us to test DNA-binding activity and affinity of TdIF1 and TdIF1 mutants. As shown in Figure 1C, the DNA-binding activity of 1–183mtN was slightly decreased (lanes 7–10) compared to that of wild-type 1–183 (lanes 3–6). While 1–183mtAT weakly bound to DNA (lanes 11–14), 1–183mtNA did not detectably bind to DNA at all (lanes 15–18). These results indicate that both the 1–75 region and the AT-hook are required for the full DNA-binding activity of residues 1–183, and that R50 and R52 are crucial for the DNA binding of residues 1–75. In a similar assay 184–329mtHTH2 lost most of its DNA-binding ability (lanes 29–32), indicating that the predicted HTH in TdIF1 functions as a DNA-binding domain. As shown in Figure 1D, mtNAH showed little capacity to bind DNA in this assay. Taken together, our results indicate that basic amino acids in residues 48–54, the AT-hook, and the HTH all contribute to TdIF1's DNA-binding activity.

### Identification of the TdIF1-binding DNA sequences by SELEX

**Figure 2:**
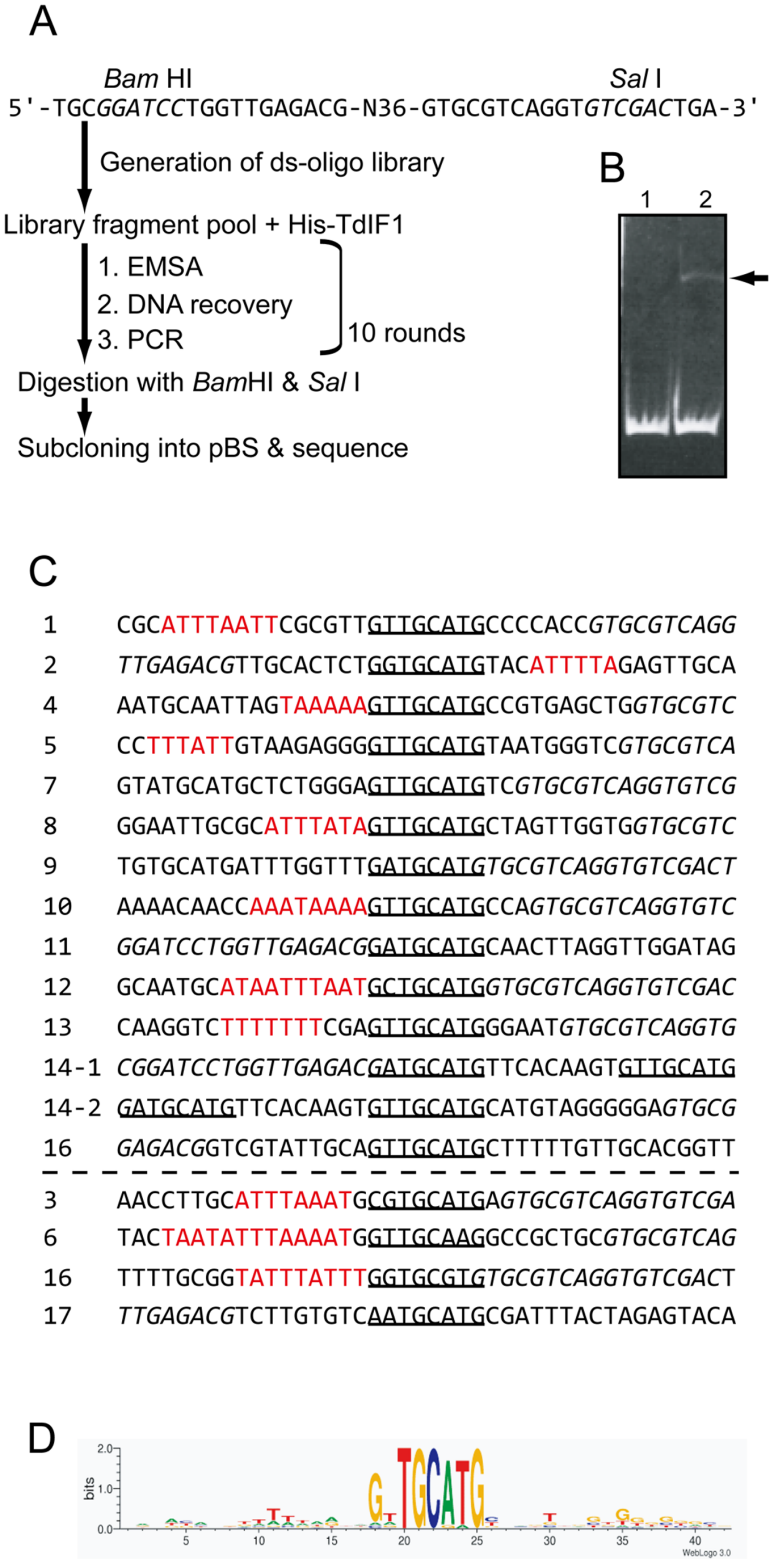
Identification of the DNA sequences recognized by TdIF1 by SELEX. (A) Flow chart of the SELEX experiment. (B) EMSA results in the 7th cycle. DNA was detected by EtBr staining. The arrow indicates the TdIF1/oligoDNA complex. (C) Oligonucleotides selected by SELEX aligned around the identified consensus 5′-GNTGCATG-3′, underlined in each case. Sequences of six or more continuous As or Ts are in red. Flanking linker sequence shown in italics. (D) Conserved nucleotides drawn as a sequence logo [20].

The AT-hook in TdIF1 preferentially binds to AT-rich sequences [5]. HTHs generally recognize specific DNA sequences; for example, the HTHs in Prrx2, Nkx3.2, and PBX1 recognize 5′-AATTA-3′, 5′-HRAGTG-3′, and 5′-ATCAATCA-3′, respectively [13], [17]–[19]. Although DNA binding of TdIF1 appears fairly promiscuous under the conditions in Figure 1, we wished to test the possibility that TdIF1 preferentially binds to a specific DNA sequence through its AT-hook and HTH. We used the random oligonucleotide binding selection strategy (SELEX) to investigate TdIF1-binding DNA sequences (Figure 2A). Purified TdIF1 was incubated with a pool of random 36-bp DNAs flanked by linker sequences (so each fragment has total length 76 bp). DNA that bound to TdIF1 was isolated by EMSA (Figure 2B) and recovered from the shifted band, then amplified by PCR and used for a second cycle of SELEX. After 10 rounds of selection, the pool of DNA fragments was sub-cloned into the pBS vector, and the DNA sequences of 17 independent clones determined.

Eleven of the 17 clones contained a continuous run of at least six As or Ts (Figure 2C, red letters), which were probably recognized by the AT-hook. Interestingly, 13 of the 17 clones contained the sequence 5′-GNTGCATG-3′ and the remaining four contained a similar sequence (Figure 2C, underlined). We aligned these sequences to produce a LOGO (Figure 2D) [20]. This analysis reveals that the HTH or residues 1–75 of TdIF1 recognize 5′-GNTGCATG-3′. Our finding of an AT-tract generally present upstream of the 5′-GNTGCATG-3′ motif suggests that TdIF1 preferentially binds to the sequence 5′-GNTGCATG-3′ following an AT-tract.

### TdIF1 preferentially binds to 5′-GNTGCATG-3′, and the binding is enhanced by consecutive AT nucleotides

**Figure 3:**
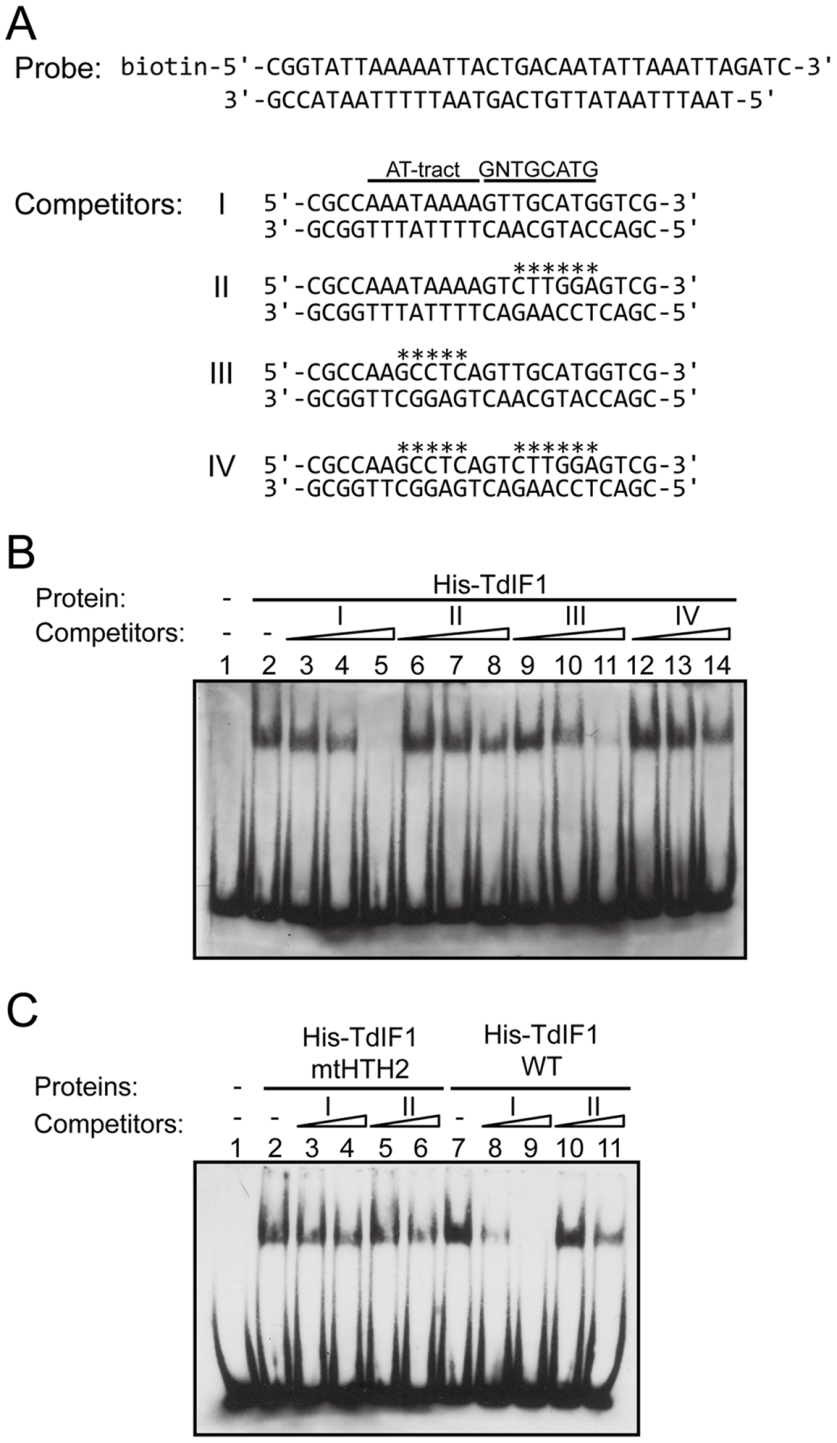
TdIF1 preferentially recognizes 5′-GNTGCATG-3′ following an AT-tract. (A) Sequences of the biotin-labelled AT-rich probe and the competitors used. Asterisks indicate replaced residues in the AT-tract or 5′-GNTGCATG-3′ motifs. (B) Competitive EMSA. All reaction mixtures contained 5 pmol of biotin-labelled AT-rich probe. Purified His-TdIF1 (100 ng) was incubated in the reaction mixture alone or with competitors (5, 15, or 45 pmol) as indicated (lanes 2–14). (C) Competitive EMSA using TdIF1mtHTH2, which has mutations in the HTH. TdIF1mtHTH2 (200 ng) or wild-type TdIF1 (100 ng) was incubated with biotin-labelled AT-rich probe alone or with competitors (15 or 45 pmol), as indicated.

To confirm that TdIF1 preferentially binds to 5′-GNTGCATG-3′ following an AT-tract, we performed competitive EMSA. We used biotin-labelled AT-rich dsDNA (biotin-dsAT) as a probe (Figure 3A), since it has been previously shown that TdIF1 can bind to the AT-rich probe in EMSA [5]. The probe contains two long AT-tracts (12 bp each) (Figure 3A), which can be recognized by AT-hook in TdIF1. To confirm that TdIF1 preferentially recognizes TdIF1 binding sequence (i.e., 5′-GNTGCATG-3′ following an AT-tract) rather than an AT-tract, we tested whether binding of the AT-rich probe to TdIF1 could be competed by unlabelled ds-oligo DNAs containing the TdIF1-binding DNA sequences (Competitor I in Figure 3A, corresponding to clone 10 in Figure 2C) or by mutated TdIF1-binding sequences (Competitors II-IV in Figure 3A). As shown in Figure 3B, the TdIF1/biotin-dsAT complex decreased with increasing amounts of ds-oligo competitor I, containing both the AT-tract and 5′-GNTGCATG-3′ (lanes 3–5), whereas the TdIF1/biotin-dsAT complex did not decrease markedly with the ds-oligo competitor II containing only the AT-tract (lanes 6-8), indicating that TdIF1 preferentially recognizes 5′-GNTGCATG-3′. The competitor II contains an AT-tract, but the competitor II did not inhibit the formation of TdIF1/biotin-dsAT efficiently (lanes 6–8). This is probably because AT-hook in TdIF1 prefers two long AT-tracts in the probe to an AT-tract in the competitor. The addition of a ds-oligo competitor III containing only 5′-GNTGCATG-3′ to the reaction mixture reduced the amount of TdIF1/biotin-dsAT complex (lanes 9–11), but its effect was slightly weaker than that of the ds-oligo containing both 5′-GNTGCATG-3′ and the AT-tract (compare lane 11 with lane 5). These results showed that TdIF1 mainly recognizes 5′-GNTGCATG-3′, and its DNA binding is moderately enhanced by a flanking AT-rich sequence.

### The HTH in TdIF1 recognizes 5′-GNTGCATG-3′

To examine whether the HTH in TdIF1 preferentially recognizes 5′-GNTGCATG-3′, we performed EMSA using purified full-length TdIF1mtHTH2, which contains intact AT-hook and the mutated HTH. TdIF1mtHTH2 bound to the AT-rich dsDNA probe, probably through AT-hook (Figure 3C, lane 2). The addition of a competitor I containing 5′-GNTGCATG-3′ reduced the amount of the wild-type TdIF1/biotin-dsAT complex (lanes 8 and 9), but did not reduce the TdIF1mtHTH2/biotin-dsAT complex significantly (lanes 3 and 4), indicating that TdIF1mtHTH2 did not recognize 5′-GNTGCATG-3′. These results collectively indicate that HTH recognizes 5′-GNTGCATG-3′, but the region containing residues 1–75 does not.

### TdIF1 up-regulates gene transcription in a luciferase reporter assay

**Figure 4:**
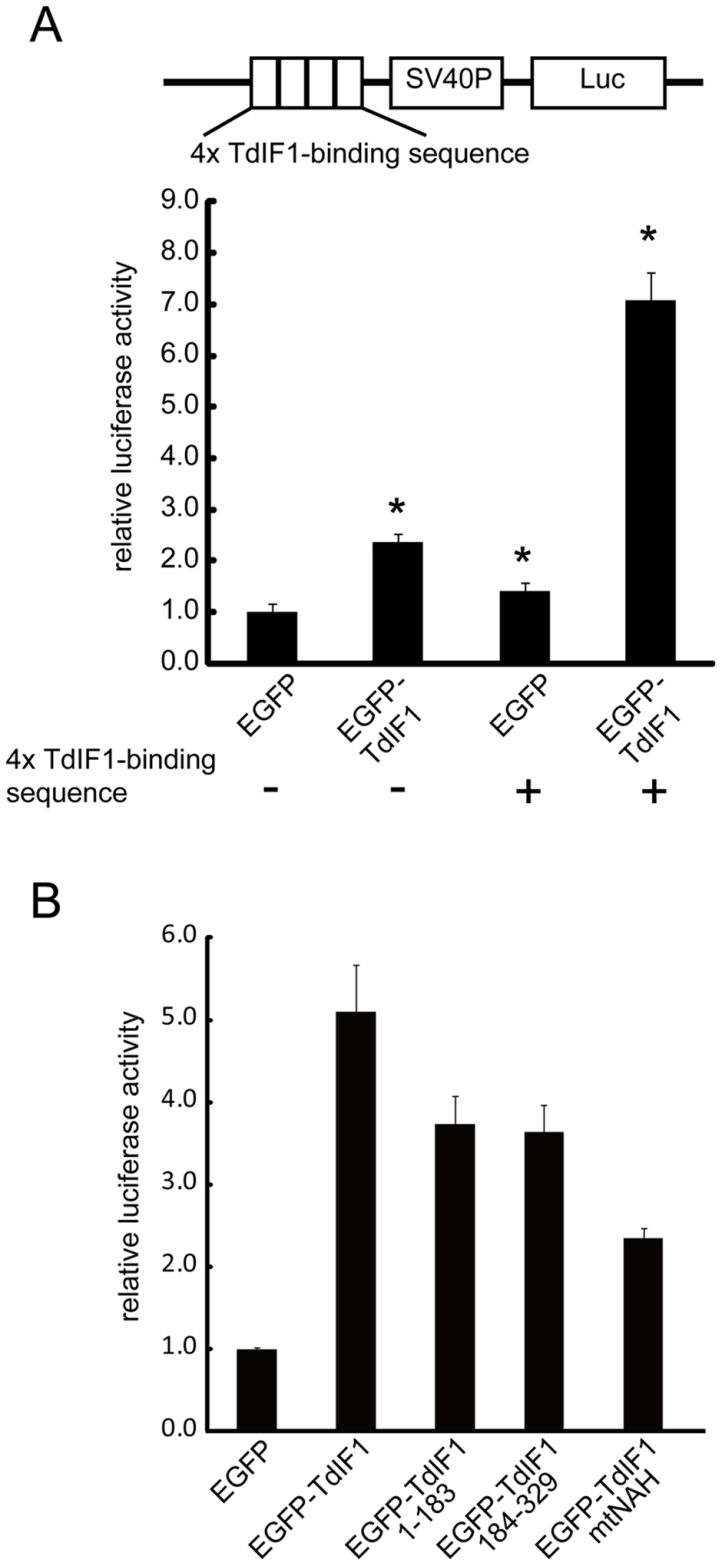
TdIF1 promotes transcription in a luciferase reporter assay. (A) TdIF1 promotes transcription. The pGL3-promoter-TdIF1-binding sequence and pRL-TK were co-transfected with pEGFP or pEGFP-TdIF1 into 293T cells, and the luciferase activity was assayed. The relative luciferase activity was normalized to the value obtained with the pGL3-promoter and pEGFP. Error bars represent S.E.M. Samples significantly different from the control are indicated by asterisks (p<0.01). (B) TdIF1 promotes transcription by directly binding to DNA. The pGL3-promoter-TdIF1-binding sequence and pRL-TK were co-transfected with pEGFP-TdIF1 truncated and point mutants, and the luciferase activity was assayed. Error bars represent S.E.M.

Since TdIF1 interacts with the transcriptional co-activator TReP-132 [4] and recognizes an AT-tract followed by 5′-GNTGCATG-3′ *in vitro*, we tested if TdIF1 is involved in gene transcription by binding to a promoter region. To examine this possibility, we performed a reporter assay in 293T cells using a plasmid harbouring four repeats of the TdIF1 DNA-binding site 5′-AAATTTGTTGCATG-3′ in the upstream-promoter region of a luciferase gene. As shown in Figure 4A (columns 1 and 3), the reporter gene transcription was increased 1.4-fold by the presence of the TdIF1-binding DNA sequence, suggesting that endogenous TdIF1 bound to the TdIF1-binding motif and promoted the reporter gene transcription. We then assayed the transcriptional activity in 293T cells transiently over-expressing EGFP-TdIF1. As shown in Figure 4A (columns 3 and 4), over-expression of EGFP-TdIF1 up-regulated the transcription 5-fold, strongly suggesting that TdIF1 functions as a transcriptional factor. Like over-expression of EGFP-fused TdIF1, over-expression of non-tagged TdIF1 up-regulated the transcription (data not shown), indicating that EGFP-tag does not affect the function of TdIF1 in transcription. Based on our analysis of its sequence preference, we propose that TdIF1 binds the 5′-AAATTTGTTGCATG-3′ sequence to up-regulate reporter gene transcription.

We next determined the regions in TdIF1 required for the up-regulation of transcription. For this analysis, we performed the luciferase assay using truncated mutants of TdIF1 (EGFP-TdIF1 wild-type, EGFP-TdIF1 1–183, and EGFP-TdIF1 184–329). As shown in Figure 4B, both the N-terminal and C-terminal regions of TdIF1 up-regulated the reporter gene transcription, suggesting that each region alone could promote gene transcription, although neither individual region up-regulated the transcription as efficiently as the full-length TdIF1. We also performed the luciferase assay using the TdIF1 point mutant mtNAH (Figure 4B), which hardly binds to DNA *in vitro* (Figure 1D). mtNAH was less able to induce luciferase activity, suggesting that TdIF1 promotes gene transcription by binding to the TdIF1-binding DNA sequence. The remaining increase in luciferase activity caused by mtNAH may imply that the mutant protein retains some ability to induce transcription, or alternatively that TdIF1 also affects luciferase activity indirectly, possibly through regulation of mRNA or protein level. The latter idea is consistent with the observation that over-expression of TdIF1 caused some increase of luciferase activity even in the absence of TdIF1-binding sequence (Figure 4A, column 2).

### TdIF1 regulates transcription of the RAB20 gene

We next searched for genes with the TdIF1-binding DNA sequence in their promoter region, using the cis-regulatory element database (cisRED) program [21], [22]. We searched for 5′-GTTGCATG-3′ alone, because this sequence was most frequently detected one in our SELEX experiment (Figure 2); the AT-tract did not appear to be essential for the DNA binding of TdIF1 (Figures 2 and 3). We identified 22 genes with 5′-GTTGCATG-3′ in their promoter region (Table S2).

**Figure 5:**
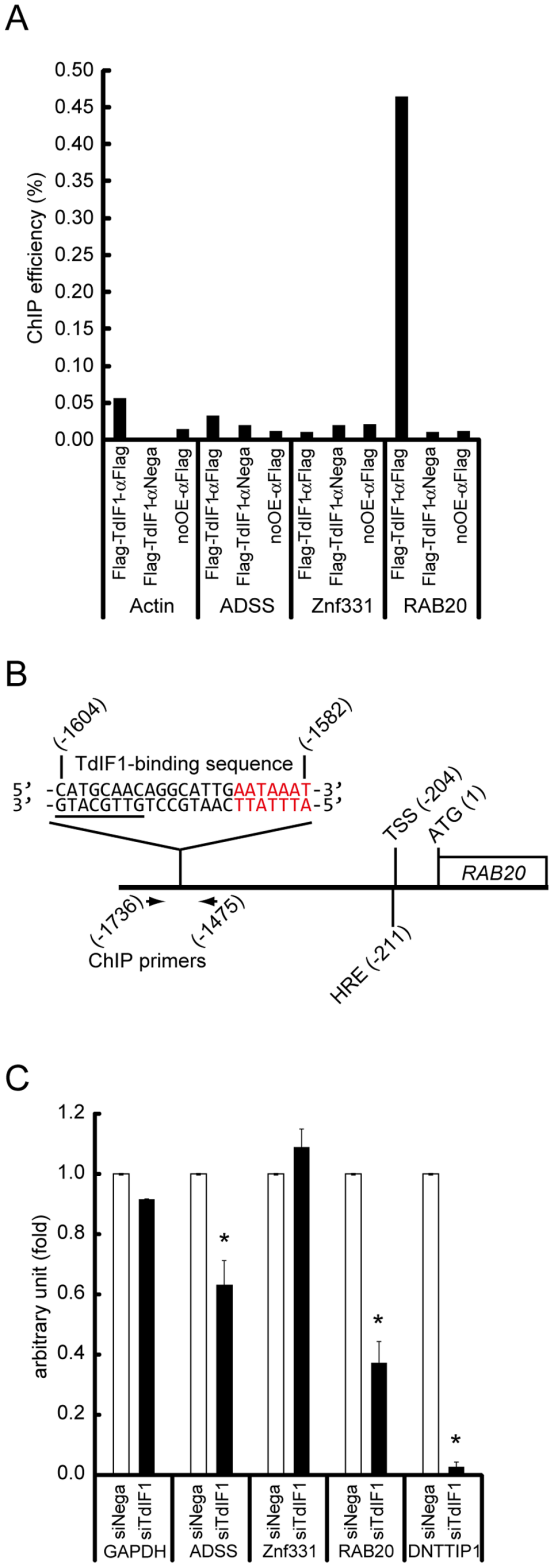
TdIF1 regulates RAB20 gene transcription. (A) TdIF1 associates with the RAB20 promoter region. ChIP-qPCR was performed using 293T cells expressing Flag-TdIF1, an anti-Flag antibody, and the indicated sets of primers (Table S1). For negative controls, an anti-myc antibody (αNega) and 293T cells not expressing Flag-TdIF1 (noOE) were used. (B) Schematic structure of the RAB20 promoter region. The motif of 5′-GTTGCATG-3′ (underlined) is positioned at -1597 bp from ATG of RAB20 gene. An AT-tract (colored red) locates near the 5′-GTTGCATG-3′ motif. Primers used in ChIP-qPCR are shown below. HRE, hypoxia responsive element; TSS, transcriptional start site. (C) TdIF1 regulates RAB20 gene transcription. Total RNA was extracted from 293T cells transfected with siRNAs against TdIF1 (siTdIF1) or negative control siRNAs (siNega). The mRNA levels were calculated by the ΔΔCt method and normalized to each mRNA from siNega. *DNTTIP1* encodes TdIF1. Error bars represent S.E.M. Samples significantly different from the control sample are indicated by asterisks (p<0.01).

We next examined whether TdIF1 actually associated with the promoter region of the candidate genes. To test this, we performed ChIP-qPCR assays using an anti-Flag antibody and 293T cells expressing Flag-TdIF1. As shown in Figure 5A, the promoter region of RAB20 was significantly amplified, with a ChIP efficiency of 0.45%. No association of TdIF1 with the ADSS or Znf331 promoter was detected (Figure 5A). While the ADSS and Znf331 promoters contain 5′-GTTGCATG-3′ only, the RAB20 promoter contains both 5′-GTTGCATG-3′ and an AT-tract (Figure 5B), indicating that the AT-tract may be essential for TdIF1's binding *in vivo*.

We then determined whether TdIF1 actually regulates RAB20 gene transcription in cells. The RAB20 mRNA in TdIF1 knocked-down cells was compared with that in control cells by quantitative RT-PCR. As shown in Figure 5C, the RAB20 mRNA decreased in the TdIF1 knocked-down cells (siTdIF1) to 37% of the level in control cells (siNega), suggesting that TdIF1 up-regulates the RAB20 gene transcription. TdIF1 knock-down was confirmed, as demonstrated by decrease in mRNA level of *DNTTIP1*, which encodes TdIF1 (Figure 5C). The mRNA level of ADSS also decreased in TdIF1 knocked-down cells (Figure 5C), suggesting that TdIF1 may also regulate ADSS gene expression. We did not detect any association of TdIF1 with the ADSS promoter (Figure 5A), but it may bind at a level below our detection threshold. Alternatively, TdIF1 might regulate ADSS gene transcription indirectly.

## Discussion

We have shown that basic amino acids in residues 48–54, the AT-hook, and the HTH are required for TdIF1 DNA-binding activity. We find moreover that TdIF1 preferentially binds to a specific DNA sequence, 5′-GNTGCATG-3′ following an AT-tract, through its AT-hook and HTH. This TdIF1-binding DNA sequence is present in the promoter of the RAB20 gene, shown here to be up-regulated by TdIF1.

The length of TdIF1's DNA-binding sequence, 5′-GNTGCATG-3′, is consistent with previous findings that HTH transcriptional factors recognize specific DNA sequences of 5-8 bps. For example Prrx2, Nkx3.2, and PBX1 recognize 5′-AATTA-3′, 5′-HRAGTG-3′, and 5′-ATCAATCA-3′, respectively [13], [17]–[19]. It is presently unclear whether the third DNA-binding region (1–75 aa) contributes to specific DNA sequence recognition. Certain arginine-rich motifs do contribute to the specific- and non-specific RNA/DNA sequence recognition, for example in RusA and Rev [23], [24]. The amino acid sequence of residues 48-52, RRGRR, is arginine-rich and similar to an AT-hook – an AT-hook being a small consensus sequence centred around a glycine-arginine-proline (GRP) tripeptide [25]. The 1–75 aa region of TdIF1 might therefore bind to an AT-tract or other specific DNA sequence. That the DNA-binding ability of the TdIF1 N-terminus is weaker than that of a typical AT-hook may be due to its sequence RRGRR, with a proline to arginine change compared to the classic AT-hook GRP sequence.

We found that TdIF1 up-regulates RAB20 gene transcription (Figure 5). RAB20 (Ras-related in brain 20) is a member of the Rab family of small GTP-binding proteins and localizes to the mitochondria or the vicinity of the Golgi apparatus [26], [27]. RAB20 gene transcription is up-regulated by Hypoxia-inducible transcription factor (HIF) under hypoxia [27]. The RAB20 promoter has a hypoxia responsive element (HRE) 7-bp upstream from the transcriptional start site (TSS) in human cells. The TdIF1-binding site is 1378-bp upstream from the TSS (Figure 5B), suggesting that TdIF1 binds to the TdIF1-binding DNA sequence to enhance transcription of the RAB20 gene promoter. It will be interesting to investigate whether TdIF1 is involved in RAB20's gene expression under hypoxia. Furthermore, RAB20 gene expression is reported to be up-regulated during the acute phase of brain inflammation [28], in pancreatic tumour cell lines, and in primary pancreatic carcinomas [26]. TdIF1 may control gene expression of RAB20 in brain inflammation and pancreatic tumour cells.

We identified the TdIF1-binding DNA sequence in several gene promoters using the cisRED program. However, we cannot rule out the possibility that the TdIF1-binding DNA sequence is present upstream or downstream of the promoter regions that we searched. When we searched for 5′-GNTGCATG-3′, instead of 5′-GTTGCATG-3′, in promoter regions using cisRED, we obtained 102 genes (Table S3). Thus, there are many more candidate TdIF1 target genes. Genome-wide mapping of the TdIF1-binding sites by TdIF1 ChIP-seq analysis should help elucidate the TdIF1-target genes, especially combined with mRNA profiling in the presence or absence of TdIF1. mRNA profiling experiments may also identify genes that are regulated by TdIF1 indirectly. These experiments are presently underway in our laboratory.

Since TdIF1 binds to a co-transcriptional factor TReP-132 [4], we suspect that TdIF1 regulates gene transcription together with TReP-132. TReP-132 is involved in the transcription of the p450scc, p21, and p27 genes [9], [29]–[33]. However, the TdIF1-binding DNA sequence does not exist in these promoter regions, implying that TdIF1 is not involved in transcriptional regulation of these genes. It is possible that TdIF1 recruits TReP-132 to promoter regions, and that the two proteins cooperate to control transcription of genes such as RAB20.

TdIF1 is reported to interact with HDAC1 and HDAC2 during M phase [10]. The HDACs usually repress transcriptional activity by deacetylating histones [34]. HDAC1 and HDAC2 interact with many transcriptional factors, including YY1, Rb binding protein-1, Sp1, and ZEB1 [35]–[40], to regulate gene transcription. Since TdIF1 functions as an enhancer of gene transcription, it is thought that TdIF1's binding to HDAC1 and HDAC2 in the M phase [10] may inhibit their deacetylase activities. Alternatively, at some specific promoters, TdIF1 may recruit HDAC1 and HDAC2 to down-regulate gene transcription in M phase.

TdIF1 was originally identified as a protein that directly binds to TdT [4], which is a member of the DNA polymerase X (polX) family [41]. PolXs are involved in DNA repair, such as in base excision repair and non-homologous end-joining [41]. Because TdIF1 also binds to the other polX family members polβ, polµ, and polλ [5], it is possible that TdIF1 acts in DNA repair by directly regulating polX family proteins. Other examples exist of transcriptional factors that are also directly involved in DNA repair, including FOXM1, KLF8, Sp1, and Tip60 [42]–[44]. Like these transcriptional factors, TdIF1 appears to function in both DNA repair and gene transcription.

## Materials and Methods

### Mutation, expression, and purification

Site-directed mutagenesis was performed by PCR using a Quick Change site-directed mutagenesis kit (Agilent Technologies). Pairs of mutagenic primers were used to construct the TdIF1 mutants, mtN, mtHTH1, and mtHTH2 (Table S1). The primers for TdIF1mtAT were described in Kubota *et al*. 2007 [5]. The substituted bases are underlined in Table S1. The nucleotide sequences were determined by the dideoxy termination method. The cDNA fragments containing each mutation were sub-cloned into the pPROEX-1 or pGEX-4T (GE Healthcare) vectors. The procedures for protein expression and purification were described previously [5].

### GST pull-out assay

The GST pull-out assay was performed as described previously [5]. Briefly, pcDNA3.1(+) (Life Technologies) plasmid digested with *Hae* III was mixed with GST-TdIF1 or GST-TdIF1 mutants bound to glutathione Sepharose 4B beads (GE Healthcare). After washing the beads, the DNA fragments bound to TdIF1 or its mutants were sequentially eluted with buffer A (50 mM Tris-HCl, pH 7.4, 5 mM β-mercaptoethanol, 0.25% Tween 20, 10% glycerol) containing 150–300 mM NaCl. The eluates were analysed on a 5% non-denaturing polyacrylamide gel, followed by silver staining.

### Generation of double-stranded oligonucleotide library

A library of single-stranded oligonucleotides 5′-TGCGGATCCTGGTTGAGACG(N)_36_GTGCGTCAGGTGTCGACTGA-3′ was generated for the systematic evolution of ligands by exponential enrichment (SELEX) experiments. The invariable 5′- and 3′-flanking sequences contained *Bam*HI and *Sal* I restriction sites. Six hundred picomoles of oligonucleotides were incubated in 100 μl of polymerase reaction buffer containing 1200 pmol of reverse primer (5′-TCAGTCGACACCTGACGCAC-3′), 0.2 mM dNTPs, and 5 units of Ex *Taq* polymerase (TaKaRa) and treated as follows: 5 min at 95°C, 20 min at 58 °C, and 20 min at 72°C. The double-stranded oligonucleotides were separated on a 12% polyacrylamide gel and purified from the gel.

### SELEX experiment

In the first TdIF1-binding reaction, 180 ng of the double-stranded oligonucleotides in the library were incubated with 100 ng of TdIF1 in a 10-μl reaction mixture containing 50 mM Tris-HCl, pH 7.4, 80 mM NaCl, 10% glycerol, and 0.25% Tween 20, for 60 min on ice. The reaction mixture was loaded onto a pre-electrophoresed 6% polyacrylamide gel, and subjected to electrophoresis at 4°C and 100 V in 0.5× TBE buffer (45 mM Tris-HCl, pH 8.3, 45 mM boric acid, 0.5 mM EDTA). The DNA was stained with ethidium bromide (EtBr). The TdIF1-bound DNA was isolated from a gel slice (∼8×5×1 mm), crushed in 200 μl of TE (10 mM Tris-HCl, pH 8.0, 1 mM EDTA)-0.1% SDS, incubated at 90°C for 5 min, and rotated at 37°C overnight. The DNA was extracted with phenol, precipitated with Ethachinmate (Wako), and resuspended in 30 μl of dH_2_O. PCR using forward (5′-TGCGGATCCTGGTTGAGACG-3′) and reverse primers was performed with 10 μl of the isolated DNA in 50 μl 10 mM Tris-HCl, pH 8.3, 50 mM KCl, 1.5 mM MgCl_2_, 0.2 mM each dNTP, 25 pmol of each primer, and 2.5 units *Taq* polymerase. PCR was performed with 10 cycles at 94°C for 30 sec, 56°C for 30 sec, and 72°C for 30 sec. The samples were loaded onto a 6% polyacrylamide gel and separated by electrophoresis at room temperature at 100 V in 0.5× TBE buffer. The DNA was extracted from the gel, phenol-extracted, ethanol-precipitated, and resuspended in 5 μl TE. Two microliters of the 5 μl of recovered DNA were used for the next round of selection. In the 2nd-10th rounds, the TdIF1 and DNA were incubated in reaction buffer containing higher concentrations of NaCl: 120 mM in the 2nd-8th rounds and 200 mM in the 9th and 10th rounds. After 10 rounds, the PCR products were digested with *Bam* HI and *Sal* I and sub-cloned into pBluescript SK (pBS; Agilent Technologies), and their DNA sequences were determined.

### EMSA

EMSA was performed as described previously [5]. Briefly, a biotin-labelled 32-bp AT-rich dsDNA (5 pmol) was incubated with purified His-TdIF1 (100 ng) in the absence or presence of competitors (5, 15, or 45 pmol) in a 10-μl final volume of binding buffer (10 mM Tris-HCl, pH 7.5, 100 mM NaCl, 1 mM EDTA, 1 mM DTT, 5% glycerol). After electrophoresis, the DNA was electrophoretically transferred to a positively charged nylon membrane, Hybond-N+ (GE Healthcare). Biotin-labelled DNA was detected using Streptavidin-HRP (GE Healthcare) and ECL reagent (GE Healthcare).

### Luciferase assay

A DNA fragment containing four repeats of the TdIF1-binding sequence was inserted at the *Bgl* II site of the pGL3 promoter (Promega). Reporter plasmids and the control plasmid pRL-TK were transfected into 293T cells. After 24 h, the cells were lysed and the *Photinum pyralis* and *Renilla reniformis* luciferase activities (PLuc and RLuc) were measured by an ARVO ×3 plate reader (PerkinElmer). The relative luciferase activity was calculated as the PLuc divided by Rluc, and normalized to the value obtained with the pGL3 promoter and pEGFP. The assay was performed in triplicate. Statistical significance was determined by Student's *t*-test.

### Quantitative chromatin immunoprecipitation-PCR

The chromatin immunoprecipitation (ChIP) assay was performed using the Low Cell# ChIP Kit (Diagenode). 293T cells were transiently transfected with pME-Flag-TdIF1. After harvesting the cells, the proteins and DNA were cross-linked in 1% formaldehyde in PBS for 15 min. The reaction was stopped with 125 mM glycine. The cells were washed in PBS and then suspended in ChIP buffer. The chromatin was fragmented with a Covaris S220 focused-ultrasonicator (duty, 20%; intensity, 5; cycles/burst, 200; at 4°C for 20 min; Covaris). After centrifugation at 12,300×g for 20 min, the fragmented chromatin was mixed with anti-DYKDDDDK-antibody (Wako)-coupled protein G-magnetic beads for 2 h. After washing the beads, the proteins were digested with Proteinase K for 30 min at 55°C. The samples were boiled and centrifuged at 12300×g for 1 min, and the purified DNA was obtained from the supernatant.

The DNA was amplified and detected using Power SYBR Green PCR Master Mix (Life Technologies) and the Applied Biosystems 7300 Real Time PCR system (step 1, 95°C for 10 min; step 2, 60 cycles of 95°C for 15°sec and 60°C for 1 min; step 3, 95°C for 15 sec, 60°C for 30 sec, and 95°C for 15 sec). The DNA was quantified using a predetermined amount of DNA as a standard, and normalized to the value of the input DNA for the ChIP assay. The DNA sequences of the primers are presented in Table S1. The set of primers for the β-actin promoter is described in Takahashi *et al*. 2006 [45].

### TdIF1 knock-down using small interfering RNA

Three TdIF1-specific small interfering RNAs (siRNAs) and negative control siRNA were designed and purchased from Cosmo Bio. 293T cells were transfected with a cocktail of siRNAs using Lipofectamine RNAiMAX (Life Technologies). The TdIF1 knock-down was confirmed by Western blotting and Real-Time PCR.

### Quantitative Reverse transcriptase PCR

Total RNA was purified from the TdIF1-knock-down or negative control cells using ISOGEN II (Nippon Gene). The cDNA was obtained by reverse transcription, amplified, and detected using a Power SYBR Green RNA-to-Ct 1-Step Kit (Life Technologies) and the Applied Biosystems 7300 Real Time PCR system (step 1, 48°C for 30 min; step 2, 95°C for 10 min; step 3, 60 cycles of 95°C for 15 sec and 60°C for 1 min; step 4, 95°C for 15 sec, 60°C for 30 sec, and 95°C for 15 sec). The DNA sequences of the primers are presented in Table S1. The DNA levels were calculated by the comparative Ct (ΔΔCt) method using glyceraldehyde-3-phosphate dehydrogenase (GAPDH) as an internal standard, and normalized to the value of the negative control. The primers for RAB20 are described in Oleaga *et al*. 2012 [46]. Statistical differences were determined by Student's *t*-test.

### Computational analysis

DISOPRED (http://bioinf.cs.ucl.ac.uk/disopred/), WebLogo (http://weblogo.berkeley.edu/), and cisRED (http://www.cisred.org/) were used.

## Supporting Information

Table S1 **Oligonucleotides used in this study.**
(DOCX)

Table S2 **Candidates of TdIF1-regulating genes having 5** ′**-GTTGCATG-3**′ **in their promoter region predicted by cisRED.**
(XLS)

Table S3 **Candidates of TdIF1-regulating genes having 5**′**-GNTGCATG-3**′ **in their promoter region predicted by cisRED.**
(XLS)
