# Supplementary material for: TdIF1 Recognizes a Specific DNA Sequence through Its Helix-Turn-Helix and AT-Hook Motifs to Regulate Gene Transcription
Source: PLoS One. 2013 Jul 10;8(7):e66710. doi: 10.1371/journal.pone.0066710 (PMC3707907; doi:10.1371/journal.pone.0066710)
Supplement: Table S1 — Oligonucleotides used in this study. (DOCX) [file pone.0066710.s001.docx]

Supplemental Table I Primers used in mutagenesis, ChIP-qPCR and RT-qPCR in this study

| Name | Method | Sequence (5’→3’) |
| --- | --- | --- |
| mtN | mutagenesis | CTGCTCGCTGCACCTGCCG |
|  |  | GCGCCCGCTCACAGATGAC |
| mtHTH1 | mutagenesis | GGTGTGGGTGCGCGATGTAGATTC |
|  |  | GAATCTACATCGCGCACCCACACC |
| mtHTH2 | mutagenesis | CGCGATGTAGATTGCTCCTGCGGTGCCCCCCATC |
|  |  | GATGGGGGGCACCGCAGGAGCAATCTACATCGCG |
| DNTTIP1 | RT-qPCR | ACTGAACGTGCGAGACAATGT |
|  |  | GCTCATGGGTCAATCTGGGTATT |
| GAPDH | RT-qPCR | CCTCCCGCTTCGCTCTCT |
|  |  | GCTGGCGACGCAAAAGA |
| RAB20 | RT-qPCR | CGCCTTCTACCTGAAGCAGTG |
|  |  | GCCGGTGATTCACATCATAG |
| ADSS promoter | ChIP-qPCR | CCGATTTTATGCTGTTTGTACTGCG |
|  |  | GCCTACCCGCTCTACCTCTC |
| RAB20 promoter | ChIP-qPCR | GCCTTTGCTTTCCTTGCCTCTC |
|  |  | CCAGGCGAAGGTGGAGTC |
| Znf331 promoter | ChIP-qPCR | CATGGCTCCATTAGGGCCGTG |
|  |  | GCATGCAACTCTACACGGCGC |
| Actin promoter | ChIP-qPCR | TGCCTAGGTCACCCACTAATG |
|  |  | GTGGCCCGTGATGAAGGCTA |
